# Rebound hypothermia after cytoreductive surgery with hyperthermic intraperitoneal chemotherapy (CRS-HIPEC) and cardiac arrest in immediate postoperative period: a report of two cases and review of literature

**DOI:** 10.1515/pp-2020-0126

**Published:** 2020-08-27

**Authors:** Sohan Lal Solanki, Mrida A. K. Jhingan, Avanish P. Saklani

**Affiliations:** Department of Anesthesiology, Critical Care and Pain, Tata Memorial Hospital, Homi Bhabha National Institute, Mumbai, India; Gastro-Intestinal Services, Department of Surgical Oncology, Tata Memorial Hospital, Homi Bhabha National Institute, Mumbai, India

**Keywords:** cancer, cardiac arrest, cytoreductive surgery, hyperthermic chemotherapy

## Abstract

**Objectives:**

Cytoreductive surgery and hyperthermic intra-peritoneal chemotherapy (CRS-HIPEC) for peritoneal malignancies are complex surgeries marked with hemodynamic perturbations, temperature fluctuations, blood loss and metabolic disturbances in the intra-operative and post-operative period. In this report, we highlighted perioperative factors which may have led to cardiac arrest in immediate postoperative period and subsequent successful resuscitation in two patients with high volume peritoneal cancers who underwent CRS-HIPEC.

**Case presentation:**

Both patients had a similar clinical course, characterized by massive blood and fluid loss, metabolic derangement, hemodynamic instability, long duration of surgery, post HIPEC rebound hypothermia and hypokalemia which need to be anticipated.

**Conclusions:**

We reviewed the literature related to postoperative hypothermia and other major complications after CRS-HIPEC and correlated the available literature with our findings.

## Introduction

Cytoreductive surgery and hyperthermic intraperitoneal chemotherapy (CRS-HIPEC) is an established treatment modality for peritoneal tumors, both primary and metastatic in origin [1], [2]. The peri-operative period is marked with varying blood loss, hemodynamic, and temperature fluctuations, electrolyte and acid-base imbalances and coagulation abnormalities and can be a challenge for perioperative physicians [3], [4]. In addition, during the HIPEC phase, these patients are treated with heated chemotherapeutic agents instilled in the abdominal cavity, predisposing them to the deleterious effects of hyperthermia along with the potential toxicity of the chemotherapeutic drugs [4], [5].

During CRS, intra-operative fluid losses may be as high as 8–12 mL/kg/h in addition to the ongoing blood loss [3], [6]. Due to the extensive nature of the surgical resection, blood and ascitic fluid loss, fluid exudation and coagulation abnormalities, the amount of fluid required to maintain normovolemia is increased [7]. This increased fluid requirement extends well into the post-operative period, as drain output continues to be high due to the severely wounded and exuding peritoneal surfaces. In addition, there is significant protein loss which is a key component of this fluid [7].

Perioperative hypothermia poses significant clinical implications of its own, leading to a threefold increase in incidence of adverse myocardial events [8]. It has been hypothesized that hypothermia induced hypertension increases plasma norepinephrine levels in the elderly which can increase cardiac irritability and predispose the patient to develop ventricular arrythmias [8]. In addition, there is a significant increase in blood loss and blood transfusion requirement, coagulopathies, wound infection, chances of prolonged post-operative recovery, and mild hypokalemia [8], [9]. Literature is limited about the effects of immediate post-operative hypothermia on patients undergoing major surgical procedures such as CRS-HIPEC and their effect on the deranged internal milieu as indicated by the hemodynamic instability encountered.

We would like to report two cases of CRS-HIPEC with similar perioperative course, wherein both patients had cardiac arrest in the immediate post-operative period and were successfully resuscitated. This report and review of literature aims to identify perioperative factors that led to the cardiac arrest and thus prevent similar occurrences of such life threatening complications in future patients posted for CRS-HIPEC.

The consent for publication of data has been taken from both patients and this data has been correlated with available literature for better understanding of the mechanism and consequences of postoperative hypothermia and its possible interactions with other major complications that occurred.

## Case presentation

Two patients were posted for CRS-HIPEC for malignant peritoneal mesothelioma and pseudomyxoma peritonei respectively. Both patients were assessed and optimized in the pulmonary rehabilitation and nutritional clinics in the pre-operative period apart from proper pre-operative evaluation including cardio-pulmonary evaluation and preoperative laboratory investigations. In both patients, general anesthesia with thoracic epidural analgesia (T8–T9) was used. Electrocardiography, pulse oximetry, non-invasive blood pressure, end tidal carbon dioxide, temperature monitoring along with advanced hemodynamic monitoring with invasive blood pressure, central venous pressure and cardiac output monitoring with FloTrac were used for both the patients. Anesthesia was maintained with sevoflurane in oxygen and nitrous oxide. Goal directed fluid therapy was administered, to achieve target values of mean arterial pressure (MAP)>70 mm Hg, cardiac index (CI) >2.5 L/min/m^2^, stroke volume index (SVI)>35 mL/m^2^ and stroke volume variability (SVV)<12. A target urine output of 0.5 mL/kg/h in the CRS phase, 2– 4 mL/kg/h during HIPEC and 1 mL/kg/h in the reconstructive phase was targeted. Intraoperative and postoperative parameters, blood gases and cardiac output parameters of both patients are mentioned and compared in Tables 1 and 2.

**Table 1:** Intra-operative and post-operative findings in both cases.

	Case 1	Case 2
Duration of surgery, (h)	16	19
Blood loss, (L)	3.8	9.4
Ascitic fluid loss, (L)	12	Minimal
Intra-operative transfusion requirement		
Packed red blood cell units	3	13
Fresh frozen plasma units	3	7
Single donor platelet units	0	1
Intra-operative fluid requirement, (L)		
Crystalloids	15	11.5
Colloids (albumin 4%)	3.5	4
Intra-operative noradrenaline requirement,(mcg/kg/min)	0.1–0.3	0.01–0.6
Intra-operative urine output, (L)	2.5	2.5
Intra-operative coagulation parameters: End of CRS, (s)		
PT	19.9	30.3
INR	1.48	2.34
aPTT	29.7	54
Post-operative coagulation parameters, (s)		
PT	26.0	33.4
INR	2.0	2.6
aPTT	51.3	52.5
Lowest core body temperature, (°C) before starting HIPEC	34.3	35.1
Highest core body temperature, (°C) during HIPEC	37.7	37.1
Delta temperature	3.4	2.0
Core body temperature on shifting to ICU, (°C)	34.0	34.4
Noradrenaline requirement on shifting to ICU, (mcg/kg/min)	0.3	0.16
Timing of cardiac arrest after shifting to ICU, (min)	240	45
Return of spontaneous circulation (ROSC), (min)	2.5	20
Drain output at the end of POD-1, (L)	6.3	2.7

aPTT, activated partial thromboplastin time; HIPEC, hyperthermic intraperitoneal chemotherapy; ICU, intensive care unit;INR, international normalized ratio; POD, postoperative day; PT, prothrombin time.

**Table 2:** Perioperative arterial blood gas and central venous blood gas analyses and cardiac output parameters for both cases.

		ABG						cVBG	Cardiac output monitor parameters		
		Case	pH	CO_2_ (mm Hg)	Base excess (mmoL/L)	K (mmoL/L)	Lactates (mmoL/L)	ScvO_2_ (%)	CI (L/min/m^2^)	SVI (mL/m^2^)	SVV %
End of CRS		Case 1	7.20	43.5	−11.2	3.84	4.99	85	2.5	25	8
		Case 2	7.39	34.7	−4	3.63	5.22	78.3	2.6	35	14
During HIPEC	Mid-way	Case 1	7.19	44.8	−11.1	3.75	6.18	76.2	2.5	32	7
		Case 2	7.34	39.3	−4.6	3.77	6.2	55.6	3.1	34	10
	End	Case 1	7.17	47.7	−11.1	3.47	6.01	79.1	3.6	37	15
		Case 2	7.32	39.8	−5.3	3.98	6.37	59.2	3.6	26	7
At the time of shifting to the ICU		Case 1	7.16	44	−13.1	2.99	5.39	85.2	2.1	13	27
		Case 2	7.32	22.9	−13	2.61	3.01	53.2	3.9	61	9
Post resuscitation		Case 1	7.29	24.3	−15	3.98	8.42	–	2.6	18	53
		Case 2	6.95	69.5	−17.7	–	12.59	–	4.1	69	13

ABG, arterial blood gas; cVBG, central venous blood gas; K, serum potassium; ScvO_2_, central venous oxygen saturation; CI, cardiac index; SVI, stroke volume index; SVV, stroke volume variation.

### Case 1

A 53-year-old man, American Society of Anesthesiologists (ASA) physical status I, with malignant peritoneal mesothelioma, underwent extensive resection, involving total peritonectomy, bilateral diaphragmatic stripping, mesenteric, and meso-colic omentectomy and cholecystectomy. The Peritoneal Carcinomatosis Index (PCI) was 26 and a completeness of clearance (CC) score 0 was achieved, which was followed by HIPEC with Mitomycin 20 mg and Cisplatin 75 mg at 42 °C for 60 min. The HIPEC phase was delayed due to hemodynamic instability necessitating fluid resuscitation and noradrenaline infusion. Noradrenaline requirement went up from 0.1 to 0.3 μg/kg/min by the end of surgery.

On admission to the surgical intensive care unit (ICU), patient was hypothermic (34 °C) with cold clammy extremities, heart rate (HR) 157/min, MAP 75 mm Hg and noradrenaline infusion ongoing at 0.3 mcg/kg/min. Serum potassium in arterial blood was 2.99 mmoL/L. Fluid boluses of 1.5 L crystalloid and 1 L albumin 4% were given. A bolus dose of 1 mEq/kg of sodium bicarbonate was given in view of severe worsening high anion gap metabolic acidosis (pH−7.16) on the arterial blood gas (ABG), followed by an infusion at the rate of 0.5 mEq/kg/h. After 4 h, blood pressure began to drop and 1 L crystalloid bolus was given. The patient then went into cardiac arrest, cardio-pulmonary resuscitation (CPR) was started and patient was successfully resuscitated with return of spontaneous circulation (ROSC) after 150 s. Echocardiography showed a collapsing inferior vena cava, for which 3 L of crystalloid and 1 L albumin 4% was rushed. Drain output was high in the immediate post-operative period and was serous in nature, along with deranged coagulation parameters and required aggressive fluid resuscitation including crystalloids, albumin, packed red blood cells and fresh frozen plasma. Noradrenaline support was slowly tapered and tracheal extubation was done over non-invasive ventilation (NIV) on second post-operative day (POD). He required intermittent NIV and oxygenation by high flow nasal cannula (HFNC) for 3 days and was shifted to the ward on POD 5.

### Case 2

A 36-year-old woman, case of pseudomyxoma peritonei, ASA physical status II, hypothyroid, who underwent total peritonectomy, near total gastrectomy, total abdominal hysterectomy, bilateral salpingo-oophorectomy, omentectomy, splenectomy, appendicular stump revision, loop sigmoid colostomy, and an accidental inferior vena cava rent which necessitated inferior vena cava repair, with bilateral intercostal drain placement followed by HIPEC with Doxorubicin 20 mg and Mitomycin-C 20 mg for 45 min. The PCI was 26 and a CC-0 was achieved. Noradrenaline infusion was started during CRS, requirement increasing up to 0.6 µg/kg/min during IVC repair, which was gradually tapered. Coagulopathy correction was started intra-operatively due to massive blood loss (9.4 L), abnormal coagulation parameters and oozing in the surgical field. Fluid management was goal directed as in case 1. The HIPEC phase was shortened to 45 min because of hemodynamic instability.

On shifting to the surgical ICU, patient had a HR of 93/min, MAP: 81 mm Hg, with cold clammy extremities, oropharyngeal temperature of 34.4 °C and noradrenaline infusion ongoing at 0.16 mcg/kg/min. Serum potassium in arterial blood was 2.61 mmoL/L. Subsequently, she developed tachycardia (HR 160/min), hypotension and required fluid boluses (500 mL crystalloid, 250 mL 4% albumin). After 45 min of shifting, patient had cardiac arrest, CPR was given and ROSC was attained after 20 min. Fluid resuscitation and coagulopathy correction was continued in the post-operative period including crystalloids, albumin, blood, and fresh frozen plasma. She was weaned from mechanical ventilation gradually and trachea was extubated on POD 6, required HFNC for 4 days and was shifted to ward on POD 11.

## Discussion

Since its introduction in the 1990s, HIPEC with time has become the standard of care for peritoneal mesotheliomas and appendicular, colorectal, gastric cancers with peritoneal metastases, while showing promising results in ovarian carcinomas [1]. With careful patient selection and optimal post-operative management, the morbidity and mortality associated with these surgeries has now been shown to be comparable with other major gastrointestinal surgeries such as Whipple’s procedure, with mortality rates ranging from 0.9 to 5.8% in high volume centers and major morbidity (grade III/IV) ranging from 12 to 52% [10]. Kim et al. reported an overall hospital mortality rate of 1.67% with median time to death being 41 days, infection and subsequent sepsis being the most significant cause [11].

In the peri-operative period, longer durations of surgery, higher PCI, massive blood loss with increased resuscitative fluid requirement and lower PaO_2_/FiO_2_ ratios were found to be associated with increased requirement of post-operative ventilation for >24 h and ICU stay for >5 days [5]. Increased transfusion requirements (≥6 units) have also been associated with increased hospital mortality [11]. Much research has been done to study the contributing factors increasing the risk of morbidity and mortality and have found to include patient factors such as age, performance status and hypoalbuminemia and surgical factors such as the PCI, bowel resection, diaphragmatic involvement, distal pancreatectomy, histology of tumor and surgeon’s experience [12].

The common intra-operative findings in both our cases included major fluid and blood losses, acute volume depletion, systemic inflammatory response syndrome, and subsequent hemodynamic instability necessitating vasopressor requirement, metabolic acidosis, raised arterial lactate levels, higher delta temperatures followed by post HIPEC rebound hypothermia, and low serum potassium levels which collectively may have resulted in the cardiac arrest.

Both our cases were primary peritoneal malignancies having a high pre-procedure tumor load indicated by a PCI of 26. The surgeries were of long duration (≥16 h). Hemodynamic instability necessitating noradrenaline infusion was first encountered during CRS. Metabolic acidosis encountered towards the end of CRS along with increased lactate levels (4.99 and 5.22 mmoL/L respectively in case 1 and case 2) could be explained by hypovolemia, hypoperfusion and extensive cytoreduction. Arterial lactate levels further rose to 6.01 and 6.37 mmoL/L respectively, at the end of HIPEC, and could be explained by the rise in core body temperature. At the time of shifting the patient to ICU, ABGs were consistent with metabolic acidosis (pH 7.16 and 7.32 respectively), high lactate levels (5.39 and 3.01 mmoL/L respectively) and significant base deficit (13.1 and 13 mmoL/L respectively). This could be attributed to multiple factors such as prolonged duration of surgery, effect of volume and blood loss and tissue hypoxia despite volume resuscitation including blood and blood products. Sodium bicarbonate therapy was instituted in case 1 in the ICU in view of severe metabolic acidosis on ABG (pH:7.16), it’s use being restricted to cases with severe persistent metabolic acidosis with pH≤7.1, to avoid risk of potential side effects of bicarbonate therapy including hypercapnia, hypokalemia, ionized hypocalcemia, increased lactate levels and QTc prolongation with no definitive proven benefits in terms of clinical outcome and mortality [13]. Mixed central venous oxygen saturation (ScvO_2_) was low in case 2 throughout the HIPEC and post HIPEC phase which could suggest an increased tissue oxygen demand. Although the serum potassium levels were in the normal range in CRS and HIPEC phases of surgery, at the time of shifting, both patients were hypokalemic (2.99 and 2.61 mmoL/L respectively) which may have added to the insult.

Both the patients were hypothermic in the immediate post-operative period (≤34.4 °C) and required active rewarming measures. Despite using warming devices on the upper part of the body during reconstructive phase, the extensive surgical field, removal of warm fluids from the surgical site, and abdominal wash may have caused persistent rebound hypothermia. This post HIPEC hypothermia may have been exaggerated by active cooling measures initiated prior to HIPEC phase, resulting in a subsequently higher delta temperature intraoperatively, which has also been found to be an independent predictor of post-operative morbidity after CRS-HIPEC [4]. Shorter duration of HIPEC and thus shorter exposure to warming measures in these cases may have also contributed to post HIPEC hypothermia. Mild hypothermia has been associated with an increase in systemic vascular resistance leading to peripheral vasoconstriction and an initial tachycardia which could lead to an increase in cardiac output [14]. With active rewarming measures, it is a possibility that the subsequent vasodilatation caused could have unmasked an underlying hypovolemia which further exacerbated the hemodynamic instability [15]. Relative hypovolemia caused due to vasodilatation primarily, venodilation, encountered during anesthesia has been attributed to multiple factors, such as the effect of anesthetic agents, loss of compensatory mechanisms, metabolic or respiratory acidosis, traumatic or surgically mediated inflammation and sepsis [16]. Kim et al. in their study showed an increase in one year mortality in post-operative patients having an increase or decrease in axillary temperatures (taking median temperature as 36.6 degree Celsius) on admission to the ICU [17]. In addition, other known complications of hypothermia, such as increased risks of cardiac events, perioperative hemorrhage, blood loss and transfusion requirements and infection rates, make it essential to maintain normothermia in the peri-operative period [18].

Both patients were successfully resuscitated after cardiac arrest and discharged from ICU and hospital with no neurological complications. In case 2, it took 20 min to attain ROSC and it is a possibility that the hypothermia may have had a protective effect against cerebral hypoxia. High drain (abdominal and intercostal drains) output also contributed to hemodynamic instability and required volume replacement along with thromoboelastography guided coagulopathy correction.

Non-invasive cardiac output monitoring like arterial pressure based cardiac output monitoring has been recommended and used regularly for CRS-HIPEC with high tumor load (PCI>15) or in cases of hemodynamic instability [3], [4], [19] and has shown improved outcome in terms of reduced post-operative complications and ICU stay [20]. These non-invasive cardiac output monitors help to assess volume status and predict fluid responsiveness. Any indication of hypovolemia intra-operatively was adequately treated with fluid boluses and further assessed in response to the fluid bolus, such that a uniform CI≥2.5 L/min/m^2^ was maintained throughout the surgery, indicating a seemingly adequate intravascular volume. SVI was however low and SVV was high in case 1 from end of HIPEC phase extending into postoperative period indicating either a volume depleted state or a peripherally vasodilated state due to the anesthetic agents as the cause of the hemodynamic instability. This highlight the challenges of goal directed fluid therapy in such cases, with varied cardiovascular compensatory mechanisms in response to extensive volume loss, anesthetic agents, temperature fluctuations, and ongoing inflammatory mechanisms [21] and thus limits its reliability. Volume replacement was carried out in a way to ensure that colloids along with blood and blood products were used to replace surgical volume loss and crystalloids were primarily used for maintenance, their requirement being grossly increased due to the ongoing inflammatory cascade.

In conclusion, we would like to reiterate that, patients undergoing extensive CRS-HIPEC can pose with significant peri-operative hemodynamic fluctuations due to massive blood and fluid losses. Undetected and uncorrected hypovolemia leading to hypoperfusion can lead to disastrous consequences. This hypovolemia may be masked by the hyperdynamic circulation caused during HIPEC and exacerbated by the peripherally vasodilated state after HIPEC attributed to anesthetic agents or rewarming measures carried out to treat hypothermia. It becomes imperative to maintain a state of normovolemia with adequate fluid replacement which must be goal directed, taking into consideration other factors such as hypothermia which may mask the underlying hypovolemia. Rebound hypothermia after HIPEC which is not commonly studied, can add to the already compromised hemodynamics with or without electrolyte abnormalities, and lead to disastrous complications like cardiac arrest. Reducing delta temperature intraoperatively by keeping constant normothermia can help prevent such complications.

## References

[1] Sugarbaker PH, Van der Speeten K Surgical technology and pharmacology of hyperthermic perioperative chemotherapy. J Gastrointest Oncol 2016; 7: 29–44. 10.3978/j.issn.2078-6891.2015.105. 26941982 PMC4754302

[2] Foster JM, Sleightholm R, Patel A, Shostrom V, Hall B, Neilsen B, Morbidity and mortality rates following cytoreductive surgery combined with hyperthermic intraperitoneal chemotherapy compared with other high-risk surgical oncology procedures. JAMA Netw Open 2019; 2: e186847 10.1001/jamanetworkopen.2018.6847. 30646202 PMC6484874

[3] Solanki SL, Mukherjee S, Agarwal V, Thota RS, Balakrishnan K, Shah BS, Society of onco-anaesthesia and perioperative care consensus guidelines for perioperative management of patients for cytoreductive surgery and hyperthermic intraperitoneal chemotherapy (CRS-HIPEC). Indian J Anaesth 2019; 63: 972–87. 10.4103/ija.ija_765_19. 31879421 PMC6921319

[4] Solanki SL, Bajaj JS, Rahman F, Saklani AP Perioperative management of cytoreductive surgery and hyperthermic intraoperative thoraco-abdominal chemotherapy (HITAC) for pseudomyxoma peritonei. Indian J Anaesth 2019; 63: 134–7. 10.4103/ija.IJA_825_18. 30814751 PMC6383478

[5] Balakrishnan KP, Survesan S Anaesthetic management and perioperative outcomes of cytoreductive surgery with hyperthermic intraperitoneal chemotherapy: a retrospective analysis. Indian J Anaesth 2018; 62: 188–96. 10.4103/ija.ija_39_18. 29643552 PMC5881320

[6] Gupta N, Kumar V, Garg R, Bharti SJ, Mishra S, Bhatnagar S Anesthetic implications in hyperthermic intraperitoneal chemotherapy. J Anaesthesiol Clin Pharmacol 2019; 35: 3–11. 10.4103/joacp.joacp_259_17. 31057232 PMC6495627

[7] Padmakumar AV Intensive care management of patient after cytoreductive surgery and HIPEC-a concise review. Indian J Surg Oncol 2016; 7: 244–8. 10.1007/s13193-016-0511-7. 27065716 PMC4818613

[8] Sessler DI Complications and treatment of mild hypothermia. Anesthesiology 2001; 95: 531–43. 10.1097/00000542-200108000-00040. 11506130

[9] Sessler DI Perioperative thermoregulation and heat balance. Lancet 2016; 387: 2655–64. 10.1016/s0140-6736(15)00981-2. 26775126

[10] Chua TC, Yan TD, Saxena A, Morris DL Should the treatment of peritoneal carcinomatosis by cytoreductive surgery and hyperthermic intraperitoneal chemotherapy still be regarded as a highly morbid procedure?: a systematic review of morbidity and mortality. Ann Surg 2009; 249: 900–7. 10.1097/sla.0b013e3181a45d86. 19474692

[11] Kim B, Alzahrani N, Valle SJ, Liauw W, Morris DL Treatment-related post-operative mortality after cytoreductive surgery and perioperative intraperitoneal chemotherapy. J Peritoneum (and other serosal surfaces) 2017; 2: 71–9. 10.4081/joper.2017.65.

[12] Newton AD, Bartlett EK, Karakousis GC Cytoreductive surgery and hyperthermic intraperitoneal chemotherapy: a review of factors contributing to morbidity and mortality. J Gastrointest Oncol 2016; 7: 99–111. 10.3978/j.issn.2078-6891.2015.100. 26941988 PMC4754303

[13] Adeva-Andany MM, Fernández-Fernández C, Mouriño-Bayolo D, Castro-Quintela E, Domínguez-Montero A Sodium bicarbonate therapy in patients with metabolic acidosis. Sci World J 2014; 2014: 627673 10.1155/2014/627673. PMC4227445 25405229

[14] Mallet ML Pathophysiology of accidental hypothermia. QJM 2002; 95: 775–85. 10.1093/qjmed/95.12.775. 12454320

[15] Ohri S, Tang A, Stephenson L Key topics in cardiac surgery. USA: CRC Press; 2004.

[16] Noel-Morgan J, Muir WW Anesthesia-associated relative hypovolemia: mechanisms, monitoring, and treatment considerations. Front Vet Sci 2018; 5: 53 10.3389/fvets.2018.00053. 29616230 PMC5864866

[17] Kim J, Oh TK, Lee J, Kim S, Song IA Association of immediate postoperative temperature in the surgical intensive care unit with one year mortality: retrospective analysis using digital axillary thermometers. Acute Crit Care 2019; 34: 53–9. 10.4266/acc.2019.00255. 31723905 PMC6849047

[18] Kim D Postoperative hypothermia. Acute Crit Care 2019; 34: 79–80. 10.4266/acc.2018.00395. 31723908 PMC6849040

[19] Garg R Cytoreductive surgery and hyperthermic intraperitoneal chemotherapy: fluid and temperature remain the culprit! Indian J Anaesth 2018; 62: 162–5. 10.4103/ija.ija_170_18. 29643548 PMC5881316

[20] Colantonio L, Claroni C, Fabrizi L, Marcelli ME, Sofra M, Giannarelli D, A randomized trial of goal directed vs. standard fluid therapy in cytoreductive surgery with hyperthermic intraperitoneal chemotherapy. J Gastrointest Surg 2015; 19: 722–9. 10.1007/s11605-015-2743-1. 25595308

[21] Kendrick JB, Kaye AD, Tong Y, Belani K, Urman RD, Hoffman C, Goal-directed fluid therapy in the perioperative setting. J Anaesthesiol Clin Pharmacol 2019; 35: S29–34. 10.1017/cbo9780511733253.012. 31142956 PMC6515723

